# Accuracy of Gypsum Casts after Different Impression Techniques and Double Pouring

**DOI:** 10.1371/journal.pone.0164825

**Published:** 2016-10-13

**Authors:** Stephania Caroline Rodolfo Silva, Aion Mangino Messias, Filipe de Oliveira Abi-Rached, Raphael Freitas de Souza, José Maurício dos Santos Nunes Reis

**Affiliations:** 1 Department of Dental Materials and Prosthodontics, Araraquara Dental School, Unesp—Univ Estadual Paulista, Araraquara, São Paulo, Brazil; 2 Division of Oral Health and Society, Faculty of Dentistry, McGill University, Montreal, Quebec, Canada; University of Brescia, ITALY

## Abstract

This study evaluated the accuracy of gypsum casts after different impression techniques and double pouring. Ten patients were selected and for each one it was obtained 5 partial putty/wash impressions with vinyl polysiloxane (VPS) material from teeth #13 to #16 with partial metal stock trays. The following techniques were performed: (1) one-step; two-step relief with: (2) PVC film; (3) slow-speed tungsten carbide bur and scalpel blade, (4) small movements of the tray and (5) without relief—negative control. The impressions were disinfected with 0.5% sodium hypochlorite for 10 minutes and stored during 110 and 230 minutes for the first and second pouring, respectively, with type IV gypsum. Three intra-oral lateral photographs of each patient were taken using a tripod and a customized radiographic positioner. The images were imported into *ImageJ* software and the total area of the buccal surface from teeth #13 to #16 was measured. A 4.0% coefficient of variance was criterion for using these measurements as *Baseline* values. The casts were photographed and analyzed using the same standardization for the clinical images. The area (mm^2^) obtained from the difference between the measurements of each gypsum cast and the *Baseline* value of the respective patient were calculated and analyzed by repeated-measures two way-ANOVA and Mauchly’s Sphericity test (α = 0.05). No significant effect was observed for *Impression technique* (*P* = 0.23), *Second pouring* (*P* = 0.99) and their interaction (*P* = 0.25). The impression techniques and double pouring did not influence the accuracy of the gypsum casts.

## Introduction

An accurate and dimensionally stable impression is an essential step for manufacturing well-fitting indirect restorations [1]. Among the broad range of elastomeric impression materials, the vinyl polysiloxane (VPS) stands out due to its excellent physical and chemical properties. One of them is the lowest permanent deformation [2], making it dimensionally stable for up to two weeks and able to be disinfected without loss of accuracy [3], in addition to the high tear resistance, neutral odor and taste. Although the literature [4,5] demonstrates that the optimum accuracy of an impression/cast is obtained with custom trays, the use of stock trays for elastomeric impressions is very usual [4] because it is an economic and simple procedure. In turn, some authors [5] show that the impression tray type does not affect the accuracy of final casts. Moreover, several studies [1,6–12] discuss pro’s and con’s of different impression techniques, there being no consensus among them regarding the choice of the ideal method for such a clinical problem.

In addition, oftentimes, for fabricating a fixed prosthesis, the gypsum cast must be trimmed into an individual stone die, which enables improved marginal adaptation of the crowns and bridges. Although new and more accurate techniques have been developed for making a removable die, its cutting out may result in significant dimensional change in the distance between the abutment teeth [13]. Therefore, producing multiple gypsum casts from the same impression is an option for reducing the clinical-lab sessions and the clinical adjustments (e.g. interproximal adjustment). While the stone dies are obtained from one cast, in order to achieve optimal marginal fit, the other one is preserved, maintaining the three-dimensional relationship between the abutment teeth [14].

Thus, this study analyzed the accuracy of gypsum casts obtained from different impression techniques and from double pouring of the same impression, by means of area measurements (mm^2^) in digital photography. The null hypothesis was that neither the impression technique nor second pouring would influence the accuracy of the gypsum casts.

## Materials and Methods

### Recruitment of patients

Ten patients were recruited following the criteria of inclusion/exclusion (Table 1), after approval by the Research and Ethics Committee for Human Subjects of the Araraquara Dental School (#75/11—FOAr / Unesp—Univ Estadual Paulista), and after written consent form signed by all participants.

**Table 1:** Criteria of inclusion/exclusion for recruitment of the patients.

*Criteria of Inclusion*	*Criteria of Exclusion*
Age between 18–80 years	Pregnancy
Absence of caries and/or periodontal disease in the maxillary right quadrant.	Allergic reaction known and informed of any material used.
Teeth of the right maxillary quadrant healthy or with satisfactory direct restorations.	Periodontal disease or impaired by caries / trauma / unsatisfactory restorations of the teeth of interest.
	Use of orthodontic braces.
	Concurrent or recent participation in another clinical study.

**Fig 1:**
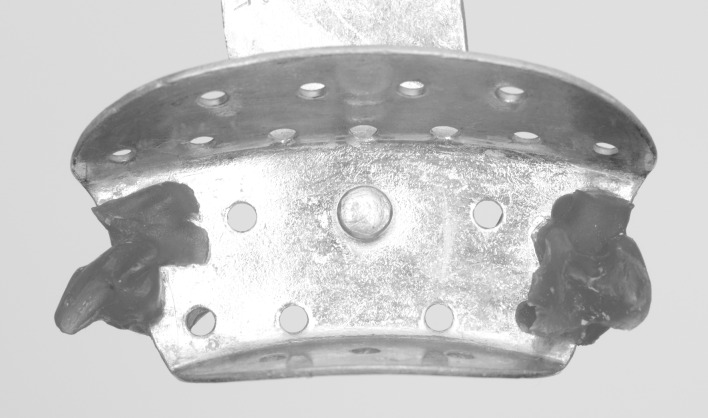
Partial metal tray customized with occlusal records in acrylic resin for each patient.

Partial metal stock trays were used for the impressions. To standardize the trays seating, position and pressure applied during impression, ensuring a homogeneous thickness of the impression material, a maxillary impression of each patient was made using condensation silicone (Speedex; Coltène/Whaledent Inc., Cuyahoga Falls, OH, USA) by the simultaneous technique. The casts were obtained by pouring vacuum-mixed type IV gypsum (GC Fuji Rock EP; GC Europe, Leuven, Belgium), following the water/powder manufacturer’s instructions, into the impression taken. The region corresponding from right maxillary canine to first molar (teeth #13 to 16) of each patient’s cast was coated with a thin layer of Cel-Lac (SSWhite, Rio de Janeiro, RJ, Brazil) and relieved with 2 sheets of wax #7 (Wilson; Polidental Indústria e Comércio Ltda., Cotia, SP, Brazil). The partial trays were placed over the wax relief and extensions were made with Pattern Resin LS (GC America, Inc., Alsip, IL, USA), by the *Nealon* technique, on the right maxillary lateral incisor incisal and second molar occlusal surfaces (Fig 1).

For each patient, 5 partial impressions were obtained from the region corresponding from right maxillary canine to first molar (teeth #13 to 16) using VPS material (Express XT; 3M ESPE, St. Paul, MN, USA). The light impression material was manipulated using auto-mixing tips, dispensing the first 3.0 cm of the mixture to ensure its homogeneity. The putty impression material was manually handled with vinyl gloves[15,16].

Ten gypsum casts in total were obtained for each patient, being two (first and second pourings) for each impression technique (Table 2).

**Table 2:** Impression techniques used for each patient.

Impression Techniques	Codes
(1) One-step	*OS*
(2) Two-step—relief with PVC film	*PVC*
(3) Two-step—relief with tungsten carbide bur / scalpel blade	*BUR*
(4) Two-step—relief with small movements of the tray	*MOV*
(5) Two-step—without relief (negative control)	*NR*

For the one-step impression technique, putty and light-body materials were manipulated and used simultaneously. For the PVC technique, a PVC film covered the putty material during the tray seating. After setting of the putty material, the impression mold was taken off from the oral cavity, and the PVC film was removed. The light material was manipulated, inserted over the putty material, and the tray was re-inserted into the oral cavity. For the tungsten carbide bur/scalpel blade technique, the impression mold was obtained with putty material and the region of the teeth was relieved with a slow-speed tungsten carbide bur (#1520; Edenta AG, Au, SG, Switzerland) during 5.0 seconds. The inter-proximal embrasures were cut with a scalpel blade n°. 15C (Swann Morton Ltd., Sheffield, England). After the relief, the putty body impression mold was relined with light material as performed for the PVC technique. For the movements of the tray technique, small horizontal movements were made during 5.0 seconds after the tray with putty material was inserted and compressed over the interested area. After material’s polymerization and tray removal from the oral cavity, the impression was relined with the light-body material as previously described.

Impressions were made by a single calibrated operator, which followed the sequence of techniques established on a blind previous raffle. The patients were randomly divided as recommended by the *CONSORT Statement*. Only one impression per day was done for each patient until all techniques had been performed. The five impressions obtained for each patient (one per technique) were carefully evaluated for retention in the tray and absence of bubbles and/or voids/delaminations. The impressions were washed in tap water for 10 seconds, disinfected by 0.5% sodium hypochlorite spray and after 10 minutes rinsed in tap water for 10 seconds. After 110 minutes, the casts were poured with vacuum mixed type IV gypsum (GC Fuji Rock EP, GC Europe, Leuven, Belgium). The casts were removed from the impression after 40 minutes, and, 120 minutes later, respecting the elastic recovery of the impression material [17,18], second pours were completed following the same procedures described above [17].

### Surface area measurement

**Fig 2:**
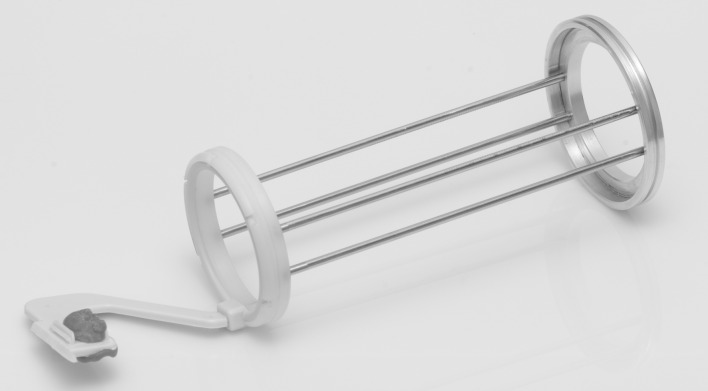
Standardizing device of each patient attached to an individual radiographic positioner with occlusal record in acrylic resin.

**Fig 3:**
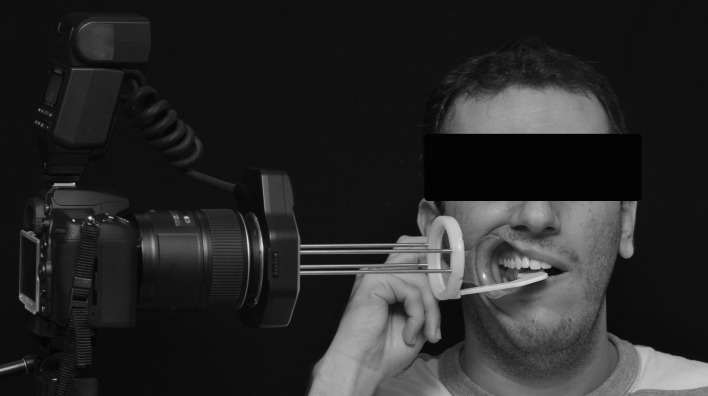
Positioning of digital camera in tripod, standardizing device and individual.

Three photographs (RAW extension; 300 dpi) of the interested region (teeth #13–16) of each patient were obtained in right lateral view using a digital camera (Nikon D7000 DLS-R; Nikon Corporation, Tokyo, Japan) with macro lens (AF-S VR Micro-Nikkor 105 mm f/2.8G IF-ED; Nikon Corporation, Tokyo, Japan) coupled to a circular flash (Sigma EM-140 DG; Sigma Corporation, New York, USA). To standardize the framing and focal length, a camera tripod was used and an occlusal bite registration in acrylic resin (Pattern Resin LS; GC America, Inc., Alsip, IL, USA) was made for each patient over a radiographic positioner, which was coupled to the camera lens (Figs 2 and 3).

**Fig 4:**
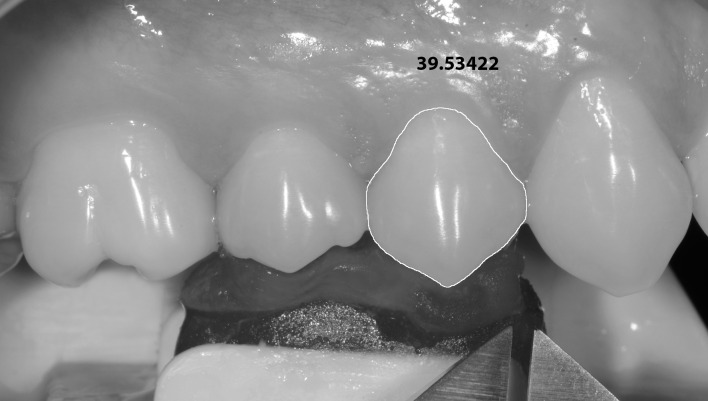
Clinical standardized photography. View of occlusal record, the ends of the digital caliper and perimeter contour of the first premolar buccal surface area, representing as the measurements were made for all teeth.

Each photograph was taken in the presence of a caliper (± 0.02 mm; Mitutoyo Sul Americana Ltda., Suzano, SP, Brazil), whose ends were fixed with opening of 1.0 mm (Fig 4). This step was used for the set scale adjustment of the *ImageJ* software (version 1.47a; NIH, Bethesda, MD, USA) by which the surface area measurements were performed. The images were imported into the *ImageJ* and a calibration was made with set scale tool. This software tool allows determining how much an object of known size has in pixels in the digital photos [10]. Thus, informing how 1.0 mm corresponds to pixels in each image, it was possible to calculate the total buccal surface area (mm^2^) of the teeth through its perimeter contouring (Fig 4). To certify the reliability of the readings, clinical photographs were taken in triplicate for each patient, and the total interested area of each image was also measured in triplicate to obtain the means and standard deviations of each experimental group. A maximum 4.0% variance was established for the acceptance and reliability of the clinical images measurements, named as *Baseline* values. Additional clinical photographs of each patient were taken after all impressions and casts were obtained. The aim of this step was to verify the absence of changes in the gingival margins in relation to their initial condition, which would influence the results, by comparing the present area values to the *Baseline*.

**Fig 5:**
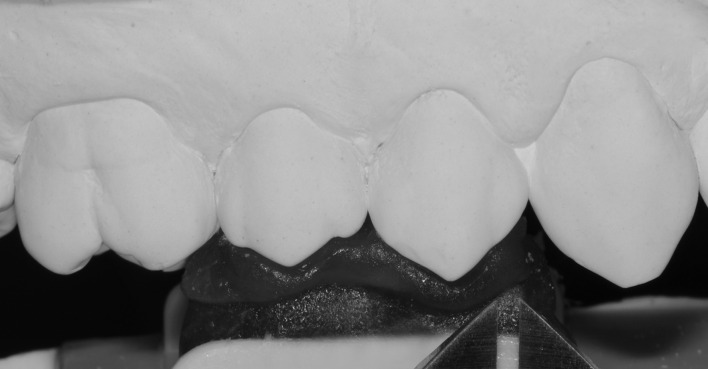
Gypsum cast photograph using the same standardization for the clinical images.

For digitization process of the gypsum casts, the same approach used for the clinical photos acquirement was used. Thus, the respective customized radiographic positioner of each patient, in the presence of the caliper with 1.0 mm opening, was again used to standardize the framing and focal length of the images taken of the gypsum casts (Fig 5). Digital images were imported into the *ImageJ* and the total interested area of buccal surface was measured. A single blinded calibrated examiner performed all measurements. The average area of each gypsum cast was compared with the *Baseline* value of its respective [19]. The differences between the *Baseline* values (clinical photographs) and those obtained from their respective casts were expressed in square millimeters.

### Statistical analysis

The data assumed normality (Shapiro-Wilk; P≥0.05) and homogeneity of variance (Levene; P≥0.05) for the sake of analysis. Statistical analyzes were applied to evaluate the influence of *impression technique* and *double pouring* on the dimensional accuracy of gypsum casts. All analyzes were performed using the IBM SPSS Statistics version 20.0 (IBM Corporation, Armonk, NY, USA).

The surface area (mm^2^) measured in each gypsum cast was subtracted from the respective area measured in intraoral images (*Baseline*) for each patient. Thus, the ultimate dimensional accuracy resulted from the difference between the dimensions calculated in the casts’ images and the dimensions calculated in the clinical images of each patient. Negative values represented higher average area of the gypsum casts in relation to the *Baseline*. Data were analyzed by repeated-measures two way-ANOVA, with *impression technique* as between-subject factor and *double pouring* as within-subject factor, and by Mauchly’s Sphericity test (α = 0.05).

## Results

All analyses used linear assumptions and respected the sphericity assumption according to the Mauchly's Sphericity test. Greenhouse-Geisser corrections were made for analyses that did not meet Mauchly's test of sphericity. After Greenhouse-Geisser correction, there was no significant effect for impression technique (P = 0.229), double pouring (P = 0.995), and their interaction (P = 0.246).

Despite the acceptance of the null hypothesis, it can be observed from Table 3 that the one-step technique, regardless of the pouring time, and the movement technique, after the first pouring, produced results with absolute values closer to those of the *Baseline* (“closer-to-zero” values). Original data are presented in S1 Table.

**Table 3:** Average values (mm^2^) from the difference between *Baseline* and gypsum casts measurements for each impression technique and pouring.

	*Double Pouring X Impression Technique*				
	*OS*	*PVC*	*BUR*	*MOV*	*NR*
*1st*. *Pouring*	-2.052	-4.863	-4.516	-1.691	-3.494
*2nd*. *Pouring*	-2.646	-5.285	-4.785	-4.563	-5.903

## Discussion

The literature has demonstrated [3,20,21] that the vinyl polysiloxanes, besides being one of the most widely used materials, is a outstanding class of impression material [5,21,22], presenting high elastic recovery (over 99%) [5], great details reproduction, high dimensional stability and excellent handling [3,20,22–24]. Its high dimensional stability and elastic recovery are physical properties of great importance when additional pours from the same mold are desired [23]; even up to two weeks [3].

Based on the obtained results, the null hypothesis was accepted, with no significant differences among the experimental groups. Negative values after the subtraction of the experimental groups from the *Baseline* were associated with the gypsum expansion, which may reach approximately 0.2% [25]. Despite the statistical equality, it can be observed in Table 3 that in some conditions the one-step (1st. and 2nd. pours) and the movement (1st. pouring) techniques showed values closer to the *Baseline*—“closer-to-zero” values. Probably, these results are related to the thin layer of the low viscosity material (light body) deposited in the interested areas in those techniques [20,26]. These findings are corroborated by other studies [9,10], which showed greater accuracy for the one-step technique (simultaneous), or no significant differences between it and relining techniques [6,7]. Fano et al. [26] studied the dimensional stability of silicone-based impression materials, reporting that as higher as the viscosity, smaller dimensional change would be expected. In turn, Hung et al. [6] described the advantages of using the one-step technique, evidencing the reduction of working time and material savings. Conversely, they stated as disadvantage the possibility of bubbles at the interface between the heavy and the light impression material. Overall, according to these authors [6], the accuracy is more affected by the material than by the impression technique. Opposite results were found by Faria et al. [10], who reported that single-phase technique with different impression materials showed higher accuracy than the two-step technique.

According to Chee and Donovan [22], simultaneous technique advocated by some manufacturers have inferior results, since the light and heavy materials are mixed simultaneously and that there is no control over the thickness and volume of material, which is normally used in excess, increasing the possibility of distortion and bubbles. Nissan et al. [21] reported that a controlled volume of the material compensates its shrinkage, with minimal dimensional changes, so that a relief space created for the light material is essential for accuracy.

This study was conducted regarding conditions closer to the dental practice. The measurements were not evaluated on a single tooth, as frequently performed [9,11,27] and the *Baseline* values were not obtained from artificial master casts/dies, as performed in previous studies [17,18,21,28]. Analyzing the area of a group of teeth, it can be expected that any minimal dimensional change be more easily detected. In addition, the dental impressions were made clinically, considering the natural emergency profile of the teeth [11], at mouth temperature, in the presence of the soft tissues and oral fluids. Other aspect that must be considered is that laboratory studies [6,8,10,12,28] frequently simulates teeth with a total crown preparation, in which retentive areas does not exist. This study was made using not prepared teeth; thus, their retentive areas were considered. Therefore, the elasticity of the impression material assumed an important role for the accuracy of the techniques. It may be presumed that the elasticity of the VPS impression material was sufficient for it removal from retentive areas, without compromising the accuracy of gypsum casts in comparison to *Baseline*, meanly when the second pouring casts were considered.

Although the present study shows positive aspects regarding the reproducibility of clinical procedures, the use of bi-dimensional evaluation and partial impressions/casts, are limiting factors, since it only considered the buccal surface area of a group of teeth. Despite these disadvantages, comparative methods of accuracy/dimensional analysis by means of digital images are easily performed, reproduced and used in literature [19,29]. Further investigations that also perform 3D analysis, in which the multidirectional distortion can be determined [30], should be conducted. In addition, although the metal stock trays are routinely used in clinical practice, future studies comparing its use with the use of acrylic resin custom trays are necessary, since some authors [22,31] consider them as gold standard for dental impressions.

## Conclusions

Based on purpose and methods used, it can be concluded that the impression techniques and double pouring did not influence the dimensional accuracy of the gypsum casts by means of surface area measurements.

## Supporting Information

S1 Table Original data values (mm^2^) from the difference between *Baseline* and gypsum casts measurements for each impression technique and pouring. Each value bellow was obtained from triplicate measurements, as explained in the manuscript.
(DOCX)
