# Supplementary material for: Accuracy of Gypsum Casts after Different Impression Techniques and Double Pouring
Source: PLoS One. 2016 Oct 13;11(10):e0164825. doi: 10.1371/journal.pone.0164825 (PMC5063298; doi:10.1371/journal.pone.0164825)
Supplement: S1 Table — Each value bellow was obtained from triplicate measurements, as explained in the manuscript. (DOCX) [file pone.0164825.s001.docx]

| **First Pouring (1st. Pouring)** | | | | | **Second Pouring (2nd. Pouring)** | | | | |
| --- | --- | --- | --- | --- | --- | --- | --- | --- | --- |
| *OS* | *PVC* | *BUR* | *MOV* | *NR* | *OS* | *PVC* | *BUR* | *MOV* | *NR* |
| -2.11 | -7.98 | -1.51 | 0.88 | -5.37 | -1.85 | -6.62 | -10.17 | 1.28 | -8.68 |
| -9.01 | -17.81 | -20.95 | -6.12 | -0.62 | -16.64 | -21.41 | -21.60 | -12.30 | -15.35 |
| -4.71 | -9.00 | -12.26 | -9.32 | -7.30 | -1.93 | -6.39 | -6.14 | -5.04 | -3.46 |
| -5.54 | 0.26 | -7.11 | 4.21 | -0.88 | -1.15 | -4.62 | 0.34 | -1.09 | -2.91 |
| -1.41 | -6.95 | 2.35 | -4.84 | -11.00 | -1.96 | -10.86 | -9.24 | -10.20 | -7.99 |
| 5.66 | 2.58 | 3.76 | 6.35 | 7.39 | 5.72 | 6.54 | 1.67 | 1.20 | 3.70 |
| 2.70 | 6.18 | 6.29 | 11.94 | 1.76 | 1.74 | 3.47 | 16.61 | 4.80 | 0.78 |
| 5.56 | -0.29 | 0.58 | -1.67 | -0.52 | 2.99 | 3.50 | -0.13 | -2.49 | -2.23 |
| -9.35 | -12.17 | -12.87 | -14.22 | -15.03 | -10.03 | -11.96 | -12.80 | -13.90 | -13.81 |
| -2.34 | -3.45 | -3.44 | -4.14 | -3.38 | -3.36 | -4.51 | -6.40 | -7.89 | -9.07 |

S1 Table- Original data values (mm^2^) from the difference between *Baseline* and gypsum casts measurements for each impression technique and pouring. Each value bellow was obtained from triplicate measurements, as explained in the manuscript.
